# Pre-operative Embolization of Primary and Secondary Bone Tumours is Safe and Effective: a Retrospective Study

**DOI:** 10.4021/wjon389w

**Published:** 2011-12-19

**Authors:** Nawfal Al-Hadithy, Panagiotis Gikas, Jonathan Perera, Will Aston, Rob Pollock, John Skinner, Kevin Lotzof, Steve Cannon, Tim W. Briggs

**Affiliations:** a Royal National Orthopaedic Hospital, Brockley Hill, Stanmore, Middlesex, HA7 4LP, UK

**Keywords:** Embolisation, Bone tumours, Blood loss

## Abstract

**Background:**

The surgical treatment of bone tumours can result in large peri-operative blood loss, due to their large sizes and hypervascularity. Pre-operative embolisation has been successfully used to downgrade vascularity, thus reducing peri-operative blood loss and its associated complications.

**Methods:**

Pre-operative embolisation was successfully undertaken on twenty-six patients with a variety of primary and secondary bone tumours.

**Results:**

Mean blood loss was 796 mL and we experienced no complications.

**Conclusion:**

Pre-operative arterial embolisation of large, richly vascular bone tumours in anatomically difficult positions, is a safe and effective method of downstaging vascularity and reducing blood loss.

## Introduction

The resection of primary and secondary bone tumours can be challenging due to their size, anatomical location and richly vascularised nature. This can cause technical difficulties including major peri-operative bleeding and its associated complications. Peri-operative blood loss for large bone tumours treated with surgery alone has had mean losses of 6000 mL, and as much as 18,500 mL [1]. This is particularly relevant in tumours where the anatomical location precludes the use of a tourniquet for vascular control, such as in the pelvis and proximal humerus, and also in renal and thyroid metastases which tends to be highly vascular large tumour masses.

Feldman et al [2] first described the use of pre-operative selective arterial embolisation to downstage the vascularity of bone tumours, thus reducing blood loss during the surgical treatment. Several studies since have shown that embolisation is an effective treatment to reduce blood loss [3-5] .

We presented of our experience with 26 patients, presenting with a variety of bone tumours (average size 10.5 x 7.5 x 5.5 cm), who underwent pre-operative arterial embolisation in our institution. These patients were referred from the regional bone tumour unit due to the nature, size or difficult anatomical location, and embolized aiming to decrease the peri-operative blood loss. The purpose of this study was to evaluate the amount of intraoperative blood loss, transfusion requirements and post-operative functional outcome.

## Patients and Methods

Twenty-six patients with a variety of bone tumours, who underwent pre-operative embolisation between 2005 and 2009, were retrospectively studied. The group comprised of 17 females and 9 males. Their mean age was 38 years old.

Embolisations were performed in the interventional suite by a senior radiologist (KL) under local anaesthesia only. Very occasionally patients required some sedation or IV analgesia, usually due to the discomfort caused by lying on the tumour for a long period of time. The procedure itself was usually not painful, unless absolute occlusion of the arterial supplied to the tumour occurred. This occurred right at the end of the procedure and was easily controlled with IV analgesia. Most vessels could be catheterized with 4 French glide catheters and occasionally microcatheters. 26 embolisations were performed with a combination of PVA particles 300-500 microns, PVA microspheres 500-700 microns, 700-900 microns, and 900-1200 microns, and Embospheres 500-700 microns, 700-900 microns, and 900-1200 microns and a variety of coils and micro coils, following selective cathererisation of individual vessels deemed to supply the tumour. The embolisation endpoint was deemed to be when all major vessels supplying the tumour had been occluded and there had been a virtual complete obliteration of tumour blush.

Surgery was performed within 48 h of embolisation. Peri-operative blood loss was measured from the suction canister, weighing swabs during surgery and from the suction drainage post operatively. Drains were removed when appropriate postoperatively and resection specimens were confirmed histologically.

Patients were scored clinically post-procedure (mean 34 months), using the Musculoskeletal Society Tumour Score (MSTS).

## Results

All 26 embolisation procedures were technically and clinically successful. The mean total blood loss was 796 mL (range 100-1800 mL), and required on average 1.1 units of blood transfusion (range 0-2).

The average MSTS functional score was 25/30 (83%).

No patient had any surgery or embolisation associated complication, and there were no wound complications, and no tumour recurred within the follow up period. All patients survived at follow up (mean 34 months). Patient demographics, histological diagnosis and anatomical site, size, blood loss, transfusion requirement, functional outcome and follow-up were shown in Table 1.

**Table 1:** Patients Demographic Data

Case	Sex	Age	Histology	Site	Size (cm)	Blood Loss (mL)	Post-Op Transfusion (units)	MSTS (March 2010)	Follow up (months)
1	F	40	Giant Cell Tumour of Bone	Left Iliac Crest	17 x 15 x 12	1200	2	24	55
2	M	19	Osteoblastoma	Sacrum	8 x 6	600	0	26	55
3	F	23	Osteosarcoma	Sacrum	4 x 3	100	0	28	55
4	M	14	Aneurysmal Bone Cyst	Sacrum	5.5 x 1.5	300	0	27	55
5	M	29	Spindle Cell Sarcoma	Left Ilium	17 x 16 x 9	1100	2	21	55
6	F	19	Aneurysmal Bone Cyst	Right Suprapubic Rami	10 x 4x 1.5	700	1	24	55
7	F	82	Haemangioma	Right thigh	8 x 7 x 5	450	1	28	55
8	M	66	Spindle Cell Sarcoma	Sacrum	9 x 4 x 2.5	300	0	26	48
9	F	61	Clear Cell Carcinoma (renal)	Right ilium	17 x 13 x 10	1500	2	25	48
10	F	23	Aneurysmal Bone Cyst	Femur	5 x 4 x 7	400	1	26	48
11	F	74	metastatic renal	Right femur	7 x 9 x 3	800	1	27	48
12	F	22	Metastatic Thyroid Carcinoma	Right Ilium	14 x 12 x 8	1800	3	22	31
13	F	25	Giant Cell Tumour of Bone	Right Ilium	18 x 16 x 7	1700	3	24	30
14	F	37	A-V Haemangioma	Right Forearm	11 x 2 x 1.5	400	0	26	31
15	F	18	Aneurysmal Bone Cyst	Right Suprapubic Rami	5 x 3 x 1	200	0	27	31
16	M	29	Haemangioma	Right Acetabulum	2.5 x 1.5 x 1	300	0	26	24
17	F	28	Haemangioma	L2 Vertebral Body	4 x 2 x 0.7	200	0	27	24
18	M	61	Clear Cell Carcinoma (renal)	Left Scapula	21 x 14 x 6	1700	2	20	24
19	F	18	Aneurysmal Bone Cyst	Radius	6 x 3 x 5	200	0	21	19
20	F	68	Giant Cell Tumour of Bone	Sacrum	7 x 9 x 3	200	0	25	19
21	M	53	metastatic renal	Tibia	12 x 10 x 5	1300	2	24	19
22	F	27	Aneurysmal Bone Cyst	Left Humerus	13 x 8 x 7	1100	2	22	15
23	M	20	Aneurysmal Bone Cyst	Ilium	16 x 13 x 9	1200	2	26	15
24	F	61	Clear Cell Carcinoma (renal)	Left femur	12 x 7 x 9	900	1	25	9
25	M	65	Haemangioma	Thigh	15 x 5 x 8	1400	3	23	9
26	F	38	Metastatic Thyroid Carcinoma	Right Ilium	8 x 8 x 3	650	1	27	9
Avg		39.2			10.5 x 7.5 x 5.5	797	1.11	24.9	34.1

## Discussion

Our results showed significantly less blood in a variety of surgically resected bone tumours as compared with the pre-embolisation era, and comparable blood loss to other studies [1-12].

The aim of embolisation is to occlude the vascular supply of the tumour. Lackman et al [6] had some success using embolisation as the sole treatment for pelvic giant-cell tumours, by downsizing and slowing the growth of tumours. However, in cases where surgical treatment is indicated, heavy, uncontrollable blood loss during surgery has been a problem. Complications arose from haemorrhage and massive transfusion requirements. Pre-operative embolisation has significantly reduced peri-operarive blood loss, and has thus allowed for more radical surgical procedures [1].

Carpenter et al [7] successfully performed pre-operative embolisations in 2 patients who had pathological fractures and encountered blood loss of < 500 mL in both cases. Bowers et al [8] reported blood loss of 450-750 mL in 6 of their 8 patients (metastatic lesions in limbs) who were successfully embolized. In the remaining 2 patients, they failed to recognize and embolize all vessels supplying the tumour and in those cases there were blood losses of 3800 mL and 7000 mL.

Salai et al [3] embolized 18 patients with large pelvic tumours (107 x 83 x 37 mm). They successfully downgraded vascularity by 2 levels (based on new tumour vascularity scale), and paid particular care to meticulously embolize all feeding vessels despite being time consuming, resulting in average blood loss of only 750 mL.

Barton et al [1], reported blood loss of 500-2800 mL in 32 patients, compared with 2000-18,500 mL (mean 6800 mL) in 20 patients operated on during the pre-embolization era.

They found that blood loss increased if surgery was not performed soon after embolisation. If surgery occurred within 3 days of embolization, blood loss was 500-1500mL, comparing to 1500-2800 mL at 4-14 days post embolisation, due to re-canalization and angiogenesis.

Chatziioannou [5] and Manke [9], both concluded that pre-operative embolisation of metastases from renal cell carcinomas was highly effective and caused a complete devascularisation of the tumour. Blood loss was reduced from 5000 mL in the control group to 1500 mL in the embolized group, and transfusion requirement was a mean of 1.69 U.

Apart from reducing blood losses by 50%, Bin Shi et al [10] found in their series of 18 patients, that pre-operative embolisation caused tumour necrosis and shrinkage, and therefore clarified tumour resection margins, allowing easier resection.

A known complication of embolization is the possibility of wound healing problems. Wirbel et al [11] noted that 2 patients in their embolization group (spinal tumours) had wound problems due to psoas muscle necrosis and subsequent skin necrosis. We noted no complications in our study.

### Conclusion

This study provides further evidence that pre-operative embolisation of tumours in difficult anatomical locations, or in highly vascular tumours keeps blood loss and its associated complications to a minimum. We experienced no complications associated with embolisations and therefore would recommend that pre-operative embolisation should be considered and used as part of the multi-disciplinary approach to the management of bone tumours in difficult anatomical locations or those that are known to be highly vascular, with large extraossesous components as seen on pre-operative imaging, such as renal and thyroid metastases.

## References

[1] Barton PP, Waneck RE, Karnel FJ, Ritschl P, Kramer J, Lechner GL (1996). Embolization of bone metastases. J Vasc Interv Radiol.

[2] Feldman F, Casarella WJ, Dick HM, Hollander BA (1975). Selective intra-arterial embolization of bone tumors. A useful adjunct in the management of selected lesions. Am J Roentgenol Radium Ther Nucl Med.

[3] Salai M, Garniek A, Rubinstein Z, Segal A, Morag B (1999). Preoperative angiography and embolization of large pelvic tumors. J Surg Oncol.

[4] Lee VN, Nithyananth M, Cherian VM, Amritanand R, Venkatesh K, Sundararaj GD, Raghuram LN (2008). Preoperative embolisation in benign bone tumour excision. J Orthop Surg (Hong Kong).

[5] Chatziioannou AN, Johnson ME, Pneumaticos SG, Lawrence DD, Carrasco CH (2000). Preoperative embolization of bone metastases from renal cell carcinoma. Eur Radiol.

[6] Lackman RD, Khoury LD, Esmail A, Donthineni-Rao R (2002). The treatment of sacral giant-cell tumours by serial arterial embolisation. J Bone Joint Surg Br.

[7] Carpenter PR, Ewing JW, Cook AJ, Kuster AH (1977). Angiographic assessment and control of potential operative hemorrhage with pathologic fractures secondary to metastasis. Clin Orthop Relat Res.

[8] Bowers TA, Murray JA, Charnsangavej C, Soo CS, Chuang VP, Wallace S (1982). Bone metastases from renal carcinoma. The preoperative use of transcatheter arterial occlusion. J Bone Joint Surg Am.

[9] Manke C, Bretschneider T, Lenhart M, Strotzer M, Neumann C, Gmeinwieser J, Feuerbach S (2001). Spinal metastases from renal cell carcinoma: effect of preoperative particle embolization on intraoperative blood loss. AJNR Am J Neuroradiol.

[10] Shi HB, Suh DC, Lee HK, Lim SM, Kim DH, Choi CG, Lee CS (1999). Preoperative transarterial embolization of spinal tumor: embolization techniques and results. AJNR Am J Neuroradiol.

[11] Wirbel RJ, Roth R, Schulte M, Kramann B, Mutschler W (2005). Preoperative embolization in spinal and pelvic metastases. J Orthop Sci.

[12] Boruban S, Sancak T, Yildiz Y, Saglik Y (2007). Embolization of benign and malignant bone and soft tissue tumors of the extremities. Diagn Interv Radiol.

